# Supplementary material for: Experience with open schools and preschools in periods of high community transmission of COVID-19 in Norway during the academic year of 2020/2021
Source: BMC Public Health. 2022 Jul 30;22:1454. doi: 10.1186/s12889-022-13868-5 (PMC9338507; doi:10.1186/s12889-022-13868-5)
Supplement: Supplementary file 1 — Additional file 1. The COVID-19 epidemic: Guide to infection control for school years 1-7.pdf. [file 12889_2022_13868_MOESM1_ESM.pdf]

The COVID-19 epidemic:

Guide to infection control for school years 1−7

20 April 2020. 4th edition 28 September 2020. Amendment 18 November 2020.

Published by the Norwegian Directorate of Health. Clinical information provided by the Norwegian Institute of Public Health and the Directorate for Education and Training

IS-2912: Guide to infection control for primary schools

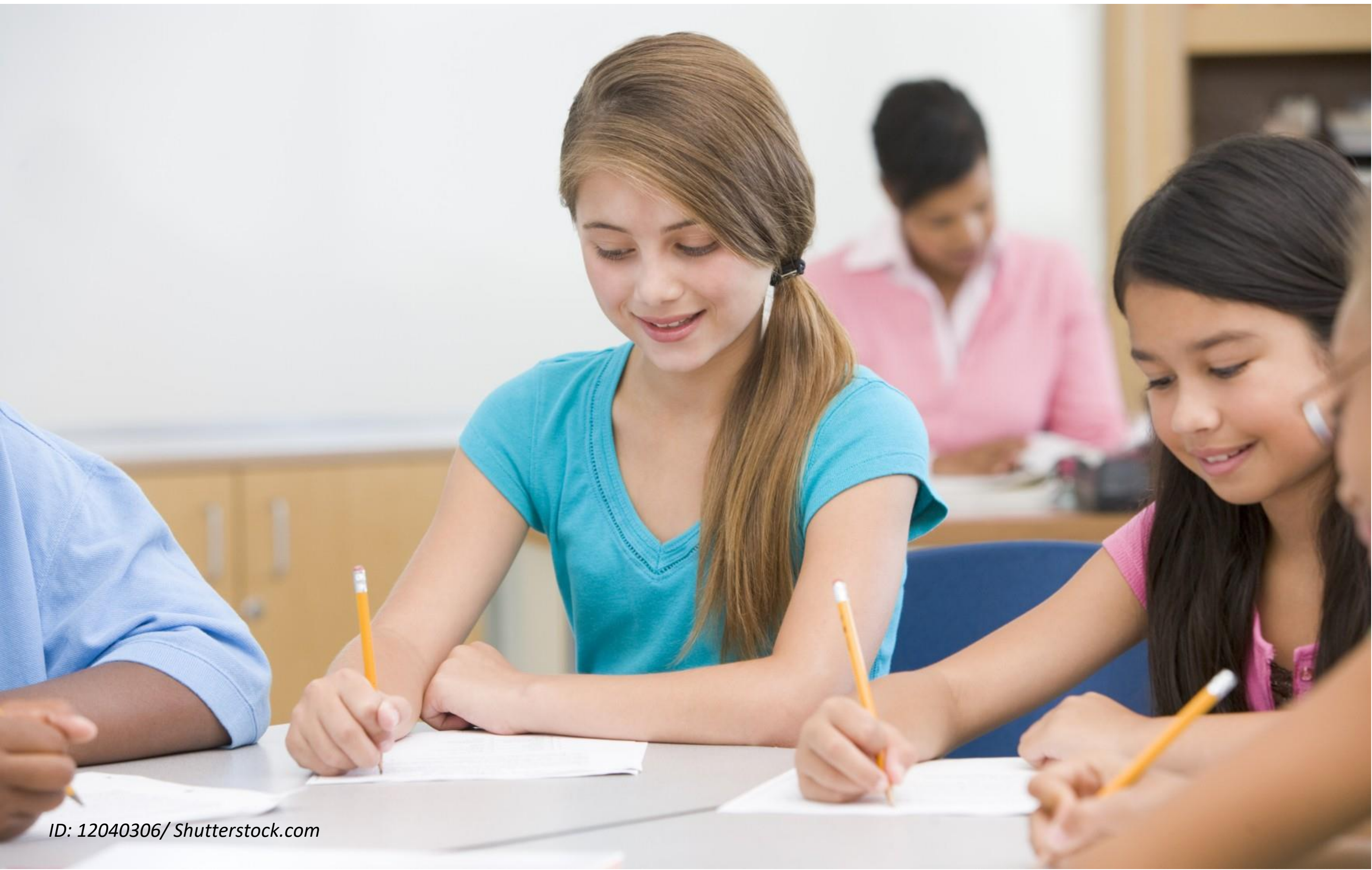

ID: 12040306/ Shutterstock.com

The COVID-19 epidemic: Guide to infection control for school years 1–7

Published by the Norwegian Directorate of Health. Clinical information provided by the Norwegian Institute of Public Health and the Directorate for Education and Training

20 April 2020. 4th edition 28 September 2020. Amendment 18 November 2020.

Contents

1 Introduction ____ 4

2 Background ____ 6

2.1 The role of schools in the community 6

2.2 About the virus, the disease and the outbreak 6

2.3 The role of children in the outbreak 7

2.4 The role of the school in outbreaks and infection tracking 7

2.5 Follow-up of vulnerable children and adolescents 7

3 Infection prevention measures ____ 9

3.1 Subdivision of infection control measures into levels (traffic light model) 9

3.2 No one who is ill should enter a school or attend after-school programmes 10

3.2.1 When should pupils and staff not attend school/after-school programmes? 11

3.2.2 When illness occurs at a school/after-school programme 11

3.2.3 What if someone in the household of a pupil/staff member is ill? 11

3.2.4 What if someone in the household of a pupil/staffmember has beenconfirmedas having COVID-19? 11

3.3 Practise good hygiene 12

3.3.1 Good hand and respiratory hygiene 12

3.3.2 Wearing of face masks 13

3.3.3 Good cleanliness 14

3.3.4 Ventilation and airing 15

3.4 Follow social distancing rules 15

3.4.1 Green level 16

3.4.2 Yellow level 16

3.4.3 Red level 18

3.4.4 Other services (applies to all levels) 21

3.5 Infection control measures in certain subjects 21

3.6 School camps and pupil gatherings across schools or municipalities 23

4 Is there anything which children and staff must pay particular attention to? ____ 24

4.1 Children with chronic diseases 24

4.2 Adults (parents/guardians/staff) 25

5 Training of staff and information for parents/guardians ____ 26

5.1 Interaction with parents/guardians and parent meetings 26

6 Visits to schools for school starters (yellow level) ____ 27

7 Checklist for infection control at schools and after-school programmes ____ 28

8 Sources ____ 30

1 Introduction

This guide gives advice and guidance to primary schools (years 1-7) which are open during the coronavirus outbreak (COVID-19). The guide is in addition to existing rules and guidelines set out in the "Regulation relating to environmental health in kindergartensand schools, etc." (*Forskrift om miljørettet helsevern i barnehager og skoler m.v.*)[1]. The development of the outbreak and practical considerations may renderit necessary for changes to be made.

It is the school owner that is responsible for ensuring that the school is managed in accordance with applicable regulations[2]. The school owner is responsible for establishing internal control procedures which ensure that the environment in the school promotes health, well-being, good social and environmental conditions and prevents illness and injury. The school owner is therefore responsible for ensuring that the school is managed in a way which addresses the need for infection control. However, it is important to stress the need for everyone to contribute to the appropriatemanagement of schools during the COVID-19 outbreak. Implementingappropriate infection control measures in a school which is open involves collaboration between all those involved in runningthe school. It requires good cooperation between the staff at the school and the school health service, as well as the cleaners. A good dialogue between the school and households will also be vital.

The guide is based around the applicableframework conditions, regulations and funding.

This guide has been prepared by the Norwegian Institute of Public Health and the Norwegian Directorate of Education and Training. The guide was prepared on behalf of the Ministry of Education and Research and the Ministry of Health and Care Services, via the Norwegian Directorate of Health.

[1] https://lovdata.no/dokument/SF/forskrift/1995-12-01-928

[2] See Section 13-10 of the Education Act (*opplæringsloven*) and Section 5-2 first paragraph of the Independent School Act (*friskoleloven*).

Change log

Amendment 18 November	• Minor adjustments of advice on school transport at yellow and red level in chapter 3.4.2 and 3.4.3.
4th edition (28 September 2020)	• The role of the school in outbreaks and infection tracking<br>• Clarification of advice for staff<br>• Advice on safeguarding of vulnerable children<br>• Advice on school camps and other gatherings<br>• Clarification of certain advice, including information concerning face masks and ventilation<br>• Deleted detailed information about symptoms, the virus and the disease, when pupils/staff can attend school, flowchart for dealing with sick people at school and vulnerable categories. Reference to the Norwegian Institute of Public Health's website for updated information<br>• Abbreviatedinformation about children and adolescents with chronic diseases and conditions. Reference to the Norwegian Society of Pediatricians' website
3rd edition (29 May 2020)	• Introduction of a traffic light model to adapt social distancing measures<br>• Improved advice regarding gentle hand washing
2nd edition (7 May 2020)	• Further explanation of cohort organisation<br>• Advice concerning the cleaning of outdoor toys<br>• Clarification of certain advice<br>• Advice for recipients of school starters

2 Background

In spring 2020, a global outbreak of the coronavirus SARS-CoV-2 led to the implementation of a series of strict measures to limit the spread of the virus through infection. All schools and kindergartens wereclosed from 13 March. The measures were reviewed on an ongoing basis, and on 7 April, the government decided that kindergartens and schools should be gradually reopened. The Norwegian Institute of Public Health (NIPH) and the Directorate of Education and Training (NDET) prepared a guide to infection control in connection with the reopening.

This guide is intended to provide an insight into and examples of how school and after-school programmes can be organised, while at the same timeensuring that infection control measures are implemented. This guide applies to all primary schools, regardless of the basis on which the school is run[3]. Regarding infection control measures relating to pupils with special needs, we also refer to the measures described in the guide for kindergartens whereapplicable.

2.1 The role of schools in the community

Schools play a crucial role in children's learning, care and development. Children and young people have both a right [4] and an obligation to attend primary and secondary education when they are likely to be in Norway for more than three months. The UN Convention on the Rights of the Child, the Norwegian Constitution [5] and the regulations within the field of education all recognise the importance of schools as an arena for children's learning, development and well-being. Schools are also vital to the structure of society, as they enable parents and guardians to go to work. School staff therefore also play a pivotal role in looking after pupils in a pandemic situation. The outbreak could last for a long time, depending on the infection control measures which are implemented within the population at large. It is therefore vital that children are able to attend school during the outbreak period, and that infection control measures are implemented covering both pupils and staff alike. To limit the spread of infection during the controlled reopening of schools, there are a number of measures that schools can implement.

2.2 About the virus, the disease and the outbreak

The disease COVID-19 is caused by the SARS-CoV-2 virus, commonly known as novel coronavirus. The virus can cause respiratory infection of varying degrees of severity. It is mainly transmitted via droplet infection, through the virus from the respiratory tract of an infected person spreading to another person via small droplets during coughing, sneezing, etc. Infection can also occur if you get droplets from your respiratory tract on your hands or objects which other people then touch (contact infection). The virus is killed by washing with soap and water and by disinfection with alcohol or temperatures above 60°C. The virus is also inactivated by sunlight.

For more information, visit the National Institute of Public Health's website.

[3] See Section 2-1 of the Independent School Act (*friskoleloven*).
[4] Right to public primary education; see Section 2-1 of the Education Act.
[5] Section 109 of the Norwegian Constitution

2.3 The role of children in the outbreak

Our current knowledge indicates thatchildrendo not play as important a role in the spreading of COVID-19 amongst the general population as adults.

Children can still become infected and experience symptoms. It is primarily children and adults with symptoms who are contagious, and the contagiousness is greatestwhen the symptoms are developing. As children tendto experience milder symptoms than adults, they are also believed to be less infectious.

There is no evidence to suggest that the infection rate is higher in kindergartens and schools than elsewhere in society. See the assessmentof the knowledge base on the Norwegian Institute of Public Health's website.

The overall negative effects of keeping schools closed are enormous. Any benefits of closing schools in order to limit infection must therefore be given particularly careful consideration. Childrenand adolescentsshould generally be the subjectof limited measures.

2.4 The role of the school in outbreaks and infection tracking

The municipal health service is responsible for following up confirmed cases of COVID-19 (infection tracking) and for notifying the school if pupils or staff test positive for the disease. The municipal health service is also responsible for determining the measures that are necessary and for defining who is a close contact of an infected person. The school's contribution is also vital:

- The school should have contingency plans in place for rapid transition to red level. This transition can be implementedrapidly if cases of infection are detected among pupils or staff, and the municipal or national authorities decide thatit is necessary to raise the level.
- The school should be able to help by providing an overview of close contacts at the school. Requirements regarding confidentiality and dataprotection must be complied with.
- The municipal health service and the school owner must work together to decide who should inform the various groups (pupils, parents/guardians, teachers and other members of staff) and how. It is important to include cover staff and temporary employees when information is disseminated, as well as people in other municipalities who are affiliated to the school where infection has occurred.
- The school must inform the municipal health serviceif any interpreters are required.
- It is important to provide everyone concerned with sufficient information, while complying with applicableconfidentiality and data protection requirements.

2.5 Follow-up of vulnerable children and adolescents

Kindergartens and schools play a pivotal role in identifying and supporting vulnerable children and adolescents. They are also important for the cooperation between the health service and the school health service, the Educational and Psychological Counselling Service (PPT), GPs, the child welfare service, mental health services, etc.

Some children and adolescents have already been identified as having special needs, while others may need special support as a result of the infection control measures. The measures can lead to social isolation as a result of both fewer opportunities for recreation and social interaction generally, and/or a lack of continuity in teaching due to quarantine restrictions or home schooling.

As a result of the current advice concerning absence in the event of respiratory symptoms, many pupils will have far higher absence rates than normal going forward. This can also occur as a result of pupils and/or staff being in quarantine. The school must find solutions which ensure that pupils who are unable to attend school receive appropriate teaching provision at home. Information about the teaching of pupils who are unable to attend school can be found on the Norwegian Directorate for Education and Training's website.

When the school or after-school programme is closed or has reduced opening hours or attendance times due to measures under the Infection Control Act, the school owner must ensure that children and adolescents with special needs have access to appropriate teaching and support during the daytime. The school owner must always consider whether pupils who receive special teaching should be offered teaching at the school. In addition, there may be pupils who cannot be accommodated when these services are closed or subject to reduced opening hours or attendance times, e.g. children living in challenging family circumstances.

It is also important to consider children and adolescents who would not be vulnerable under normal circumstances. The school owner is responsible for reviewing and assessing who should be offered support. Parents and pupils should therefore not have to request such services.

The school health service must be available to follow up children and adolescents and, in cooperation with the school, encourage pupils to contact the service themselves.

Schools must

- establish good routines for cooperation with the health service, Educational and Psychological Counselling Service (PPT), the child welfare service and other municipal services in order to obtain an overview of the children and adolescents who are in need of support for health, personal, social or emotional reasons.
- draw up plans concerning how they can ensure that children and adolescents are offered appropriate services and support.

  establish routines and cooperation with relevant services to ensure the follow-up of children and adolescents with high absence rates.

3 Infection prevention measures

Many measures are being implemented throughout society which are collectively limiting the spread of infection. It is important to use measures which are appropriate for the situation concerned. It is therefore not always possible to use exactly the same measures in schools, businesses and the population at large. Here, we describe measures to limit the spread of infection among pupils in years 1-7 and staff. It is not necessary to implement any measures over and above those described here, unless determined otherwise by local or national authorities.

The disease will continue to occur for as long as the virus is circulating in society; it is not possible to prevent every single new case. This means that infection may still entera school, but the measures described here will limit the spreadof infection among children and staff. It is important not to place the blame on particular individuals.

The aim of the advice is to limit the spread of COVID-19. The three cornerstones for slowing the spread of infection are:

1. No one who is ill should attend school.
2. Practise good hygiene
3. Follow social distancing rules

The most important infection control measure is for infected people to stay at home. Good cough etiquette and social distancing are essential for limiting droplet infection, while hand hygiene, particularly avoidingtouching your face with dirty hands, is importantto prevent contact infection. Maintaininga greater physical distance betweenpeople reduces the risk of infection, even before symptoms develop.

3.1 Subdivision of infection control measures into levels (traffic light model)

It will be necessary to adapt the infection control measures according to the local situation with regard to both infection and outbreak management as a whole. This is done for schools by dividing the measures into levels using a traffic light model with green, yellow and red levels. The measures range from the near-normal organisation of the school day (green level), to more comprehensive measures with small, fixed groups and a greater distance between pupils (red level). The differences between the three levels particularly concern the measures implemented to ensure social distancing (section 3.4).

The municipalhealthservicepossesses infection control expertiseand can be consulted regardingquestions aboutorganisation andfacilitation at local level. The infection control authorities are responsible for deciding what level themeasures should beset at according to the traffic lightmodel. Local reductions in levels from the national level are currently not permitted.

Table 1 presents the key features of the traffic light model for primary schools. More detailed information is provided below.

Table 1: Traffic light model for primary schools

Primary school years 1-7	Measures
Green level	1) No one who is ill should attend school<br>2) Practise good hygiene and normal cleanliness<br>3) Social distancing measures:<br>Avoid physical contact (handshakes and hugging)<br>Normal organisation of classes/groups and the school day
Yellow level	1) No one who is ill should attend school<br>2) Good hygiene and extra cleaning<br>3) Social distancing measures:<br>Avoid physical contact (handshakes and hugging)<br>The whole class is considered to be a cohort*<br>Staff can alternate/move between cohorts*/classes<br>Cohorts organised based on year groups* or group-based division of after-school programmes<br>Avoid congestion and crowding<br>Allocate defined areas in the playground for different classes/cohorts* during play and breaks
Red level	1) No one who is ill should attend school<br>2) Good hygiene and extra cleaning<br>3) Social distancing measures:<br>Avoid physical contact (handshakes and hugging)<br>Divide classes into smaller cohorts*<br>One teacher is dedicated to each cohort* wherever possible<br>Cohorts* in after-school programmes should be the same as in school where possible<br>Pupils and staff must maintain social distancing<br>Avoid congestion and crowding<br>Allocate designated areas for different classes/cohorts* during breaks/play to reduce contact between different cohorts*<br>Consider staggering the start and end of the school day for pupils

* In this guide, fixed groups of pupils are referred to as a 'cohort'. This term is used to highlight the fact that the purpose of the organisation is to limit infection. The size of the cohort and other social distancing measures will depend on the situation amongstthe general population. See the explanatory information in section 3.4.

3.2 No one who is ill should enter a school or attend after-school programmes

No one should attend school if they are ill. This applies at green, yellow and red levels.

The symptoms of COVID-19, particularly in children, can be mild and difficult to distinguish from other respiratory infections. It is primarily peoplewith symptoms who are contagious, and they are most contagious while their symptoms are developing. This is also why it is important to pay attention to symptoms that have developed recently, while residual symptoms following a respiratory infection are of lesser importance as regards infectiousness.

More information about symptoms, the clinical picture and when it is possible to attend school can be found on the Norwegian Institute of Public Health's website (adultshere and children here).

3.2.1 When should pupils and staff not attend school/after-school programmes?

It is important that parents/guardians and staff are notified and understand the reasons why children who are experiencing symptoms of respiratory infection should not attend school/after-school programmes.

Pupils and staff with respiratory symptoms:

- Pupils and staff with recently developed respiratory symptoms and/or general malaise must not attend school/after-school programmes, even if their symptoms are mild. The Norwegian Instituteof Public Health's websiteprovides up-to-date advice on sick adultsand children.
- They should stay at home until they have recovered. Pupils and staff can return to school when they feel healthy and free from fever, even if they have some residual symptoms of respiratory infection (some nasal mucus, blocked nose and mild cough).

Pupils or staff in isolation or quarantine:

- It is assumed that pupils, parents/guardians and staff will not go to the school if they are in quarantine or isolation. The school is not responsible for identifying who needs to go into isolation or quarantine. For more information, visit the National Institute of Public Health's website.

3.2.2 When illness occurs at a school/after-school programme

It is important that pupils who experiencesymptoms of respiratory infection are sent home (after their parents/guardians have been notified) or are collected as soon as possible. Sick pupils should not travel on public transport. Pupils who are collected must wait in a separate room together with a member of staff or outside where there are no other children around. Where possible, the member of staff should keep at least two metres away, but a balance must be struck in relation to the age of the pupil and their care needs. If it is not possible to maintain a distance of two metres and the pupil is comfortable doing so, the pupil should put on a face mask to reduce the spreading of infection. If the pupil does not wish to wear a face mask, the adult should put on a mask, unless a distance of two metres can be maintained. Anyone who has been in contact with the pupil must remember to wash their hands afterwards. The room, toilet and other areas where the pupil has been must be cleaned afterwards. It is sufficient to use ordinary detergents.

Staff members who fall ill while at school/after-school programme musttravel home as soon as possible. Sick staff members should not travel on public transport. Sick staff members should wear a face mask to reduce the spreading of infection if they are unable to maintain a distance of two metres away from other people.

3.2.3 What if someone in the household of a pupil/staff member is ill?

If someone in their household is experiencing the symptoms of respiratory infection but has not been confirmed as having COVID-19, the pupil/staff member mustattendschool as normal. The pupil may also attend after-school programmes.

3.2.4 What if someone in the household of a pupil/staff member has been confirmed as having COVID-19?

If someone in the household has been confirmed as having COVID-19, close contacts must be kept in quarantine in accordance with the advice issuedby the health authorities. No one who is in quarantinethemselves mustattendschool or after-school programmes.

3.3 Practise good hygiene

3.3.1 Good hand and respiratory hygiene

Hand washing and good cough hygiene are important at green, yellow and red levels.

Good hand and cough respiratory hygiene (cough etiquette) reduce infection in connection with all respiratory infections, including COVID-19 infection. These measures should reduce infection via objects and hands, and reduce infection via coughing. Ensuringthat your hands are clean is the most important measure thatyou can take to prevent such indirect infection.

Hygiene measures mustbe carried out frequently by everyone, regardlessof what they know about their infection status or that of other people around them. It is important that all pupils and staff are familiar with the measures below. As regards the youngest pupils, procedures can be established whichthe childrenfind enjoyable to follow. Hand washing should generally take place with an adult present, particularly in the case of the youngest pupils.

With regard to pupils with special needs, e.g. where nappy changes are necessary, see the guide for kindergartens.

Hand hygiene:

Good hand hygiene should be practisedin the right way, at the right time and using effective and gentle products. Good facilities and good procedures are essential for compliance.

Pupils and staff must wash/disinfect theirhands:

- When arriving at school
- Before meals/handling food
- After going to the toilet
- When their hands are visibly dirty (e.g. when coming in with visibly dirty hands after playing outdoors)
- When moving from one classroom to another
- After contact with bodily fluids (e.g. after blowing your nose)
- After contact with animals

Both hand washing and alcohol-basedhand disinfection are usually effective. Hand washing with soap and water is normally recommended. Hands can be dried using disposable paper towels or an air dryer.

Alcohol-based hand disinfectants can readily be made available and are kind to the skin. They can therefore be a good alternative to hand washing, but theyare less effective on wet or visibly dirty hands. It is not necessary to carry out both hand washing and hand disinfection, as this can lead to skin irritation. Either is sufficient.

If your hands are visibly dirty and no soap and water are available, you can use wet wipes to wash your hands, possibly followed by hand disinfection (e.g. when you are outdoors).

Table 2: How should hand hygiene be practised.

**Hand washing**	**Hand sanitiser**
• Moisten your hands in lukewarm water<br>• Apply soap (unperfumed, pH <5.5)<br>• Rub your hands together to spread the soap so that it covers all hand surfaces<br>• Rinse your hands well<br>• Pat-dry your hands using paper towels (do not rub your hands together; this will make you hands sore)<br>• Turn off the tap using a disposable paper towel	• Apply hand sanitiser to your hands<br>• Spread the sanitiser well on both hands and gently rub into the skin<br>• Once your hands are dry, they are clean
**Skincare:**<br>You should moisturise your skin when necessary. If using shared tubes, make sure that the opening of the tube does not come into contact with skin or objects during use so that the cream does not become contaminated.	

For more advice on hand hygiene, including product selection, procedures and skincare, see the Norwegian Institute of Public Health's website.

Cough hygiene:

- It is recommended that paper towels be provided in a readily accessibleplace for use by pupils/staff. If paper towels are not available, it is recommended thatpupils/staff cough or sneeze into their elbow when necessary.
- Avoid touching your face or eyes. This can be difficult for young children, but older pupils and staff should be encouraged to follow this advice.

3.3.2 Wearing of face masks

The wearing of face masks at school is not recommended for either pupils or staff. However, the school should have face masks available for use if pupils or staff fall ill and it is not possible to maintain a safe distance. See the advice on the Norwegian Institute of Public Health's website.

It is not recommended thatpupils in 7th grade and younger(under 12-13 years) wear a face mask in the current infection situation, even in areas with a rising infection rate. This is in line with the recommendations issuedby the WHO. This is because there is no evidence to suggest that children play an importantrole in the spreading of infection, and that children find it more difficult to wear a face mask correctly.

It is recommended that staff members who are at greater risk of developing severe COVID-19 symptoms contact their GP and employer if their workplace needs to be adapted (see section 4.2).

3.3.3 Good cleanliness

3.3.3.1 Green level

At green level, normal cleaning will be sufficient. Novel coronavirus (SARS-CoV-2) is easily removed by manual cleaning using waterand ordinary detergents.

3.3.3.2 Yellow and red level

More thorough and frequent cleaningis recommendedat yellow and red level. The school owner should review procedures and local cleaning plans, and make any necessary adjustments (organisation, responsibility and resource requirements).

The virus can survive on surfaces for anything between a few hours and a few days, depending on the type of surface, temperature, sunlightand other factors. Thorough and frequent cleaning is therefore important in order to prevent infection.

Extra cleaning is important in exposed areas:

- Toilets and washbasins must be cleaned at least daily.
- Disposable paper towels and soap should be provided and refuse bins must be emptied regularly.
- Dining tables must be cleaned after use using water and soap.
- Door handles, stair banisters, chairs, other tablesurfaces and other items thatare often touched must be cleaned frequently, at least daily.
- Desks/workplaces must be cleaned daily; it can be a good idea for pupils to do this themselves.
- Toys, tablets, computers/keyboards which are sharedmust also be cleaned, at least daily.
- Reduce the number of toys in order to make cleaning more feasible.
- Toys and equipment which are only used by one cohort need not be cleaned daily.
- As an alternative to daily cleaning, toys and equipmentcan also be tidied away ("quarantined") for two to three days before they are reused. Class teddy bears, etc. may be used if they can be washed in a washing machine betweenhome visits with pupils, or if they are given a "rest break" of 2-3 days between each pupil.
- Tableware and cutlery must be washed in a dishwasher in accordance with the applicable procedures.

It is not necessary to use additional protective equipment whencleaning. Wash hands after cleaning has been carried out, even if gloves have been worn.

It is not necessary to use disinfectants routinely at school. However, if disinfectants areused, visible dirtmust first be wiped off usinga cloth or paper towel; otherwise, thedisinfectant will not work. Alcohol- andchlorine-based disinfectants may be used.

It is not necessaryto cleanoutdoorplay equipment or toys whichare used outdoors. The most importantconsideration is to washyour hands before andafterplaying outdoors, and before eating. Thevirusis also inactivatedby sunlight.

Changes of clothing mustbe keptin the changing rooms in accordance withnormal routines.

3.3.4 Ventilation and airing

In general, good ventilationshouldbe ensuredin enclosedenvironments andsealedrooms. Sealed rooms with no ventilationsystem shouldbe airedregularly. Existingventilation systems should otherwise beusedand maintained inthe normal way.

3.4 Follow social distancing rules

Social distancing will reduce the risk of infection from people who do not know they are contagious. The overriding goal of social distancing measures is to limit the spread of infection between people and to limit the numberof contacts. To achieve this, you can increase the distance between people and/or reduce the number of people who are close together.

It is important to stress that staff will continue to attend to their pupils' needs regarding contact and care.

Major events must be organised in accordance with the applicable guidelines issued by the Norwegian Institute of Public Health and the Directorate of Health.

About cohorts

As regards pupils at primary school, where it can be difficult to control the distance they maintain from each other, the most important measure will be to limit the number of close contacts of each pupil and staff member by having fixed groups (cohorts). Reduced contact between cohorts will also limit the spread of infection. It will also make the task of infection tracking easier and prevent the whole school from having to close when a pupil or staff member is confirmed as being infected.

More contact is permitted betweenchildrenthan between adults because they do not play such an important role in the spreading of infection.

Cohort sizes can be adapted to local circumstances, the size of the class and the individual school's circumstances. Cohorts must have as little interaction withother cohorts as possible. Within a cohort, pupils and staff can socialise and play together.

Two cohorts can work together if necessary for practical purposes duringthe day and so that staff can have a break. Such cooperation can take place outdoors where possible (joint outdoor teaching, play, excursions and sports).

Cohorts which are not working together can take it in turns to use outdoor areas and the areas need not be cleaned in connection with each changeover. Cohorts which are not working together but are outdoors at the same time should preferably use separate outdoor areas.

Staff

Staff members should keep at least one metre away from other members of staff throughout the working day. If a member of staff becomes infected, measures mustthen be implementedto ensure that other staff members do not become infected or have to go into quarantine, and to ensure that absencelevels do not impact on teaching provision. The school's management must considerhow many staff members may be present, depending on the floor area that is available in offices, communal areas, etc. See also the Advice for workplaces for advice on infection prevention in the workplace.

Schools are encouraged to limit contact between staff members who do not naturally work together, wherever possible. The use of assistants, part-time teachers and specialist personnel can represent a particular challenge in that, through theirrole, they often come into contact with both pupils and other staff members across classes and grades.

3.4.1 Green level

Physical contact:

- The need for closeness and care must be considered. Employees mustwash their hands after close contact and comforting.
- Shaking hands, hugging and unnecessary physical contact must be avoided wherever possible.

Organisation of cohorts

- Pupils do not need to be divided into cohorts.

Limit the sharing of food and items:

- Pupils must not share food and drink.

Specifically for staff:

- Avoid handshakes and hugging.

3.4.2 Yellow level

The overriding goal at yellow and red levels is for pupils and staff to have a limited number of contacts and to keep track of them.

Physical contact:

- The need for closeness and care must be considered.
- Shaking hands, hugging and unnecessary physical contact must be avoided wherever possible.

Organisation of cohorts:

- A class is considered to be a cohort.
- In the case of open-plan schools, pupils should be divided into groups corresponding to the size of a normal class.
- Staff can alternate between cohorts to teachin thenormalway.
- The composition of cohorts canonly be alteredafter eachweekend.
- Two cohorts can work together for practicalreasons during the day. Cooperation can take place outdoors where possible.
- Indoors, cohorts not working together may pass each other andremain in thesame room for limited periods of time (up to 15 minutes). However, cohorts may remainin the same room for longer periodsof time, providedthereis a minimum distanceof two metres betweenthem at all times.
- Cohorts (which are not workingtogether) shoulduse separate outdoors play areas. Either they can take it in turns to use the outdoor areas, or the outdoor areas can be divided into zones. The outdoor areas need not be cleaned.
- After-school programmes should be organised in groups to continue to limitthe total number of contacts. Dependingon the numberof pupils, this can bedone by maintaining the cohorts, division into other groups, or organisation based on year groups.

Density in groups:

- Pupils should preferablyhavetheir own permanent desk/workplace.
- It is not necessaryto limitthenormal movements of pupilsaround the classroom.
- Large gatherings shouldbe avoided.
- All children should have their own place for eating and activities during afterschool programmes.
- Avoid congestion on the way in and out of classrooms and in changing rooms and toilets.
- Alternatively, apply markings to the floor to ensure that a safe distance is maintained between pupils in group rooms/classrooms, changing rooms and other areas that can become congested.
- Make greater use of outdoor time and outdoor schooling, including during afterschool programmes.

Play and breaks:

- Arrange for additional adults to be out at break times in order to help pupils maintain a safe distance from each other.
- Consider scheduling breaks at differenttimes to limit the number of pupils who are out at the same time (depending on the number of pupils and the size of the playground).

Limit the sharing of food and items:

- Pupils must not share food and drink.
- Food can be prepared at school/after-school programme in accordance with normal guidelines. There is no evidence to suggest that COVID-19 infection can be passed through food.
- Children should eat in their cohorts. In the case of shared canteens, cohorts should eat at different times. Tables and chairs must be cleaned after each group.
- Limit the sharing of stationery, tablets and other equipment.
- Textbooks can be taken between home and school, but not shared between pupils. This also applies to written work which is handed in.
- Toys should not be brought from home.

Transport and school transport:

- The use of public transport to and from the school should be limited as much as possible.
- When using public transport, pupils should maintainsocial distancing from each other.
- School trips that involve gatherings of pupils in groups or on public transport should be limited.
- School transport: Pupils who rely on school transport may take a bus or other means of transport to get to and from school. Follow the current national or local guidelines for public transport. Pupils from the same household or cohort can sit together.
- Pupils must maintain a safe distance from each other while waiting for the bus or other means of transport.
- Local assessments may be made in consultation with the local healthauthorities.

Specifically for staff:

- Avoid handshakes and hugging.
- Maintain a safe distance (at least one metre) from colleagues in all situations.
- Where different teachers mustbe used, teachers can switch between cohorts, while the cohorts remain in fixed classrooms wherever possible.
- Limit the use of public transport duringrush hour to and from the workplace wherever possible. Follow local recommendations concerning the wearing of face masks.
- Consider holding digital meetings instead of physical meetings where possible.
- In the event of staff absence, temporary cover staff may be required or staff may need to be reassigned betweencohorts. This can be done as and when necessary, but reassignments shouldbe limitedas much as possible.
- Permanent or temporary staff who work at a number of schools must undergo training concerning the applicable local procedures that apply at each school. Temporary staff should avoid working in different schools on the same day.

3.4.3 Red level

The overriding goal at yellow and red levels is for pupils and staff to have a limited number of contacts and to keep track of them.

Pupils should ideally not socialise with many other children in their spare time.
Physical contact:

- The need for closeness and care must be considered.
- Shaking hands, hugging and unnecessary physical contact must be avoided wherever possible.

Organisation of cohorts:

- Create small cohorts: The size of cohorts should be based on the ratio between teachers and pupils in accordance with the standard requirementregarding teacher-pupil ratios[6] (15 pupils per teacher in years 1 to 4, and 20 pupils per teacher for years 5 to 7).
- Cohort sizes can be adapted to local circumstances and the size of the class:
    - For practical reasons, it is possible to increase the size of a cohort size by a few extra children.
    - Schools must themselves considerwhether it is prudent to increase the size of cohorts based on the age of the pupils, the level of activity, the need for adult supervision and the ability to maintain a safe distance between pupils.
- As a general rule, one member of staff should accompany their cohort.
- Staff from cohort 1 can provide relief in cohort 2, and vice versa.
- Cohorts should move between classrooms as little as possible.
- Two cohorts can work together for practicalreasons during the day. Cooperation can take place outdoors where possible.
- The composition of cohorts can only be altered after each weekend.

[6] See the Regulation pursuant to Section 14A-1 of the Education Act.

- Indoors, cohorts not working together may pass each other and remain in the same room for limited periods of time (up to 15 minutes). However, cohorts may remain in the same room for longer periods of time, provided there is a minimum distance of two metres betweenthem at all times.
- Cohorts (which are not working together) should use separate outdoors play areas. Either they can take it in turns to use the outdoor areas, or the outdoor areas can be divided into zones. The outdoor areas need not be cleaned.
- Cohorts should also be maintained duringafter-schoolprogrammes. Dependingon the number of pupils, other solutions maybe adopted locally whichlimit the overallnumber of contacts.

Specifically for years 5 - 7:

- Staff members should go to the classroom, so that pupils do not need to move to another room.
- Pupils and staff should follow social distancing rules wherever possible, including within cohorts, preferably one metre.

Density in groups:

- All pupils should have their own desk in the classroom to ensure that social distancing is maintained betweenchildren. All pupilsshould have their own desk in the classroom to maintain a safe distance between children (preferably one metre).
- It is not necessaryto limitthenormal movements of pupilsaround the classroom.
- All children should have their own place for eating and activities during afterschool programmes.
- Avoid congestion on the way in and out of classrooms and in changing rooms and toilets.
- Alternatively, apply markings to the floor to ensure that a safe distance is maintained between pupils in group rooms/classrooms, changing rooms and other areas that can become congested.
- Make greater use of outdoor time and outdoor schooling, including during afterschool programmes.
- Use larger premises wherevernecessary and possible.
- Locally, it may be appropriate to consider a school and the associated after-school programme in context, in order to facilitate greater flexibility in terms of the timing of lessons and after-school programmes, thereby reducing the group size of children.
- In the event of a shortage of physical space at the school, consideration can be given to the use of alternating days, staggeredattendancetimes or alternative premises.
- Major events must be avoided

At the start and end of the day:

- Agree dispersed places wherepupils can assemble before the start of the school day in order to limit the number of pupils gathered together in one place and to avoid congestion on the way in and out of classrooms.
- Parents/guardiansmust not accompany their children onto the school premises or into changing rooms unless absolutely necessary. If parentshave to come to the school, they must keep their distance from other parents, children and staff.

Play and breaks:

- Arrange for additional adults to be out at break times in order to help pupils maintain a safe distance from each other.
- Consider scheduling breaks at differenttimes to limit the number of pupils who are out at the same time (depending on the number of pupils and the size of the playground).
- For example, schedule breaks for one or two year groups at a time. This must be assessed based on the number of pupils in each year.

Limit the sharing of food and items:

- Pupils must not share food and drink.
- Food may be prepared at school/after-school programmes in accordance with normal guidelines. There is no evidence to suggest that COVID-19 infection can be passed through food.
- Children should eat in their cohorts. In the case of shared canteens, cohorts should eat at different times. Tables and chairs must be cleaned after each group.
- Limit the sharing of stationery, tablets and other equipment.
- Educational materials, toys, etc. should not be shared between cohorts until theyhave been cleaned.
- Textbooks can be taken between home and school, but not shared between pupils. This also applies to written work which is handed in.
- Toys should not be brought from home.

Transport and school transport:

- The use of public transport to and from the school should be limited as much as possible.
- When using public transport, pupils should maintainsocial distancing from each other.
- School trips that involve gatherings of pupils in groups or on public transport should be avoided.
- School transport: Pupils who rely on school transport may take a bus or other means of transport to get to and from school. Follow the current national or local guidelines for public transport. Pupils from the same household or cohort can sit together.
- Pupils must maintain a safe distance from each other while waiting for the bus or other means of transport.
- Local assessments may be made in consultation with the local healthauthorities.

Specifically for staff:

- Avoid handshakes and hugging.
- Maintain a safe distance (at least one metre) from colleagues in all situations.
- Where different teachers mustbe used, teachers can switch between cohorts, while the cohorts remain in fixed classrooms wherever possible.
- Limit the use of public transport duringrush hour to and from the workplace wherever possible. Follow local recommendations concerning the wearing of face masks.
- Use digital meetingsinstead of physical meetings wherever possible.
- Shared tablets/computers/keyboards must be cleanedafter use.

- In the event of staff absence, temporary cover staff may be required or staff may need to be reassigned betweencohorts. This can be done when necessary, but reassignments shouldbe limitedas much as possible.
- Permanent or temporary staff who work at a number of schools must undergo training concerning the applicable local procedures that apply at each school. Temporary staff should avoid working in different schools on the same day.

3.4.4 Other services (applies to all levels)

Special services

- Special services (Educational and Psychological Counselling Service (PPT), speech therapists and others) must follow basic infection control procedures in accordance with the applicable recommendations (good hand and respiratory hygiene, do not go to work when ill, etc.), but can otherwise perform their work in the normal way.

Specifically for the school health service:

- Public health nurses play an important role as advisers regarding infection control at schools. Public health nurses are also important collaboration partners for pupils and with regard to the provision of health-related and psychosocial supportin accordance with national guidelinesfor health centres and the school health service.
- Teaching, discussions, surveys, monitoringand vaccination of pupils can be carried out according to the same guidelines as for other health and care services. See the Norwegian Institute of Public Health's website.

School libraries

- Books may be loaned provided that hand washing is practised before arrival at the school library.
- The number of visitors is determined by the ability to maintaina safe distance between those who do not normally belong to the same cohort.
- Return of books following illness. Under all circumstances, pupils withsymptoms of respiratory infection must stay at home until they have been symptom-free for 24 hours, or longer if they are in quarantine or isolation. Books can therefore be returned when the child themselves is able to return to school.

3.5 Infection control measures in certain subjects

Some advice is given below regarding specific assessments whereadditional measures are required, in addition to the recommendations given above. Unless stated otherwise, no additional measures are recommended.

Green level

- Teaching can take place in the normal way

Yellow level

- Physical education:
    - The use of outdoor gyms is recommended whenever possible
    - Changing rooms and showers may be used in the normal way (within cohorts)

  - When teaching takes place in premises that do not belong to the school, the entire cohort may be taught together (e.g. in a public swimming pool or sports hall) and the group size need not be limited to what is recommended in the guide for sports.

- Music:
  - Avoid the sharing of wind instrumentsbetween several pupils.
  - In connection with the use of handheld instruments and keys, surfaces that are touched must be wiped off after use. If shared equipmentmust be used by a number of pupils, hand washing is recommended before and after use.
  - Pupils may sing in choirs within their cohort. There is no reliable evidenceto indicate that singing in choirs can increase the risk of infection.
- Food and health:
  - There is no evidence to suggest that COVID-19 infection can be passed through food.
  - Teaching can be conducted in the applicable cohorts.

Red level

- Physical education:
  - The use of outdoor gyms is recommended whenever possible.
  - A cohort may use changing rooms and showers provided they do not become crowded. If necessary, a cohort can be split.
  - If appropriate, changing prior to PE lessons may take place in different classrooms in order to provide more space between cohorts.
  - It is recommended that showering after physical exercisebe deferred until the pupils have returned home.
  - Avoid activities involving close contact between pupils.
  - When teaching takes place in premises that do not belong to the school, the entire cohort may be taught together (e.g. in a public swimming pool or sports hall) and the group size need not be limited to what is recommended in the guide for sports.
  - Swimming lessons: The chlorine in swimmingpool water inactivates both coronaviruses and other viruses. Teaching must be organisedso as to avoid close contact between pupils in changing rooms and the pool.
- Music:
  - Instruments and other musical equipmentshould not be shared or touched by anyone other than the person who will use it. If shared equipmentmust be used by a number of pupils, hand washing is recommended before and after use.
  - Avoid the sharing of wind instrumentsbetween several pupils.
  - In connection with the use of handheld instruments and keys, surfaces that are touched must be wiped off after use.

   - Pupils may sing in choirs within their cohort. There is no reliable evidenceto indicate that singing in choirs can increase the risk of infection.
   - When singing together or playing wind instruments together, it is recommended that pupils stand at least one metre away from each other.
- Food and health:
   - There is no evidence to suggest that COVID-19 infection can be passed through food.
   - Teaching can be conducted in the applicable cohorts.
- Arts and crafts
   - Limit the sharing of equipment. Clean equipment after use wherever possible.

3.6 School camps and pupil gatherings across schools or municipalities

School camps bring together pupils from differentregions. Where applicable, these schools should also follow the "Advice for pupils living at a boarding school" in the guide for lower and upper secondary schools. Pupils should live and take part in activities within their cohort. School camps should not be arranged in municipalities which are at the red level. Similarly, if a class/school is at red level, the pupils concerned should not attend a school camp.

At pupil gatherings/vocational teaching where pupilsfrom several schools in the same municipality gather together, provision should be made to ensure that pupils who do not belong to the same cohort can maintain the recommended distance.

In the case of special national or regional educational initiatives where pupils gather together over several days, the “Advice for pupils living at a boarding school” in the guide for lower and upper secondary schools should be followed where appropriate.

Provision should be made to ensure that safe distances can be maintained both in and outside teaching situations. Participants should be organised into smallergroups in order to limit the number of contacts. At red level, it is recommended thatprofessional meetings be conducted digitally.

For both school camps and other pupil gatherings, there must be a plan for dealing with pupils who fall ill or have to go into quarantine. This involves ensuring that facilities are available for isolation/quarantine and thatroutines are in place for travel home.

Appropriate procedures must be established for information, both in advance of gatherings and in the event of illness or infection. Consideration must be given to whether the local health authorities shouldbe contacted in advance of a school camp/gathering to ensure that any cases of infection can be managed.

4 Is there anything which children and staff must pay particular attention to?

4.1 Children with chronic diseases

Children and adolescents rarely become seriously ill from novel coronavirus. This also applies to children and adolescents who already have chronic diseases or conditions. However, some children with a serious illness or condition may be advised not to attend school as a precaution.

In consultation with the Norwegian Institute of Public Health, the Norwegian Society of Paediatricians has preparedan overview of various diagnoses in children and adolescents and any special considerations which should be taken into account. The Norwegian Society of Pediatricians believes that the vast majority of children with chronic diseases or conditions can and should attend kindergarten or school.

Children and adolescents witha chronic disease/condition who **can attend school** in the normal way, include:

- Children and adolescents withdiabetes
- Children and adolescents withwell-controlled asthma
- Children and adolescents withallergies
- Children and adolescents withepilepsy
- Children and adolescents with Down syndrome
- Children and adolescents withheart defects without heartfailure
- Children and adolescents withan autoimmune disease who use immunosuppressive therapy and have a stable condition
- Former premature children withoutsignificantlung disease
- Children and adolescents withobesity

Categories of children and adolescents withone or more chronic diseases/conditions, where consideration can be given on an individual basis to whether or not there are grounds for the child/adolescentto be kept at home and/or receive facilitated teaching from school, are listed on the Norwegian Society of Pediatricians' website.

The school and parents/guardians should discuss whether it is possible to arrange for these pupils to attend school. When assessing how the teaching and special provision can be carried out, considerable emphasis must be placed on what would be in the best interests of the pupil[7]. For example, it will be crucial that the benefits of receiving facilitated education at home outweighthe disadvantages to these young people as a result of losing out on important social and academic development. In cases where there is a need for facilitated teaching, this must be clarifiedwith the attending paediatrician.

As a general rule, there is no basis for siblings of these children and adolescents to be kept at home.

[7] See Article 3 of the Convention on the Rights of the Child

4.2 Adults (parents/guardians/staff)

Some groups are at greater risk of developing a more severe form of the disease, but most people even in the vulnerable categories only experience mildsymptoms. As we have learned more about COVID-19 and the associated risk factors, the advice on who belongs to the vulnerable categories has gradually become more nuanced. The age groups and diseases that may increase the risk level for adults are regularly updated on the Norwegian Institute of Public Health's website.

Staff belonging to groups at greater risk of developing severe COVID-19 symptoms should be assessed individually in relation to adaptedwork. Staff being considered for such work must have a medical certificate.

Parents/guardianswho are at riskcan contact their GP to assess the need for facilitating school provision/home education in order to limit the risk of infection in the home. For pupils with parents/guardians (others in the household) who are at risk, a dialogue should be established between the school and the parents/guardians concerning whether it is possible to facilitate the teaching so that these pupilscan still attend school in the normal way. In such cases, consideration for the child's best [8] interests should also be afforded great importance.

[8] See Article 3 of the Convention on the Rights of the Child

5 Training of staff and information for parents/guardians

Staff must be trained to carry out the necessary infection control measures described in this guide. The guide will be supplemented withadditional information which can be used in the training of staff.

Parents and guardians mustbe confident that it is prudent for their childrento return to their school/after-school programme. It is therefore important that the school has a good dialogue with parents and guardians. It is also important that parents and guardians actively contribute to implementingthe infection control measures described in this guide. Specific information is being prepared for this group.

For more information on this, see the Norwegian Directorate for Education and Training's website.

5.1 Interaction with parents/guardians and parent meetings

Parents meetings and other events organised by schools are considered to be public events and must be conducted in accordance with the current recommendations for events issued by the Norwegian Institute of Public Healthand the Directorate of Health.

At red level, we recommend the use of digital meetings. Physical parents meetings and other larger gatherings are not recommended.

Personal appraisals can be conducted at green and yellow levels, subject to normal infection control advice. At red level, the use of digital meetings is recommended. However, face-to-face discussions in the form of physical meetings may be necessary. In such cases, the ordinary infection control advice must then be followed.

6 Visits to schools for school starters (yellow level)

School starters come from different kindergartens. This leads to the mixing of children and parents/guardians and requires special consideration. The same key principles of infection control as described above must be applied (no infected persons present, good hygiene, and reduced contact between individuals).

Organisation:

- Depending on the number of school starters each school has, the number of school starters, including parents/guardians, who can be present at the same time should be determined in advance. Consideration must be given to the current nationalguidance for events, group sizes and gatherings issued by the Directorate of Health.
- If sufficient space is available, several groups of people may be present at the same time, but in different places.
- The children should be divided into smaller groups
- The children must not have close physical contact with godparents in higher years.
- Where possible, children from the same kindergarten should attend at the same time. Consideration can be given to whether the children can attendschool in their cohorts together with kindergarten staff.
- The children should have their own desk in the classroom.
- If parents/guardians must accompany children, they mustalso be able to keep at least one metre away from other children and staff.
- Meetings with parents/guardians shouldbe conducted in accordance with the current national guidance for events, group sizes and gatherings issued by the Directorate of Health.

7 Checklist for infection control at schools and after-school programmes

A checklist is given below covering the measures that must be in place. The measures are described in more detail above. The school owner is responsible for ensuring that infection control measures are in place, and that staff are familiar with the content and implementation of infection control measures[9].

Measures	Date carried out	Remarks
**The school owner's overriding responsibilities**		
Draw up contingency plans for rapid transition between different levels of measures in accordance with the traffic light model		
Train staff regarding infection control measures by familiarising them with the information given in this guide		
Information for parents/guardians concerning new routines at schools/after-school programmes		
Prepare plan for hand washing procedures for pupils and staff		
Prepare written procedure for cleaning		
Prepare plan for establishment and organisation of cohorts		
Establish dialogue with any staff who are in a risk group and children who require special provision		
Draw up plans for safeguarding vulnerable pupils		
**Hygiene measures**		
Make sure sufficient soap and paper towels are available at all handwashing stations and toilets		
Training of pupils in handwashing procedures and cough etiquette		
Put up posters about handwashing procedures and cough etiquette		
Provide alcohol-based disinfectants where no handwashing facilities are available		
Plan hand hygiene measures to be applied outside or on excursions (wet wipes and alcohol-based disinfectants)		
**Cleaning (yellow and red levels)**		
Draw up a cleaning plan, which describes the frequency and methods to be used.		

[9] See Regulation No. 470 of 27 March 2020 relating to infection control measures, etc. regarding the coronavirus outbreak

Measures	Date carried out	Remarks
Draw up a plan for cleaning toys, tablets, etc. Toys and items that cannot be cleaned must be tidied away		
**Follow social distancing rules (yellow/red level)**		
Consider the use of rooms in relation to the number of pupils in the cohorts. Use larger premises if possible		
Assess outdoor activities, including staggered times for different cohorts		
Divide outdoor areas so that pupils from different cohorts do not mix insofar as is possible		
Avoid large gatherings of pupils		
Ensure that sufficient stationery and other equipment/materials is available to limit sharing		
Provide a separate desk/chair per pupil with a safe distance between pupils		
Provide a separate seat for each pupil during meals and activities, with a safe distance between pupils		
Ensure good routines for meals		
Plan to reduce congestion in changing rooms, toilets and on the way into and out of the premises		
If appropriate, apply markings to floors to ensure safe distances are maintained in areas where congestion may occur		
Plan for alternating times for breaks to limit the number of pupils who are outside at the same time		
Plan for additional adults to be out at break times in order to help pupils maintain a safe distance from each other		
Plan dispersed places where people can assemble before the start of the school day in order to avoid congestion		
Plan school transport		
Avoid using public transport for trips out of school		
**For staff**		
Limit physical meetings; arrange digital meetings if possible (yellow/red level)		
Maintain the recommended distance from other staff members in all situations		
Limit use of public transport		

8 Sources

Tiltak på skole- og barnehageområdet under koronautbruddetvåren 2020, rapport 03.04.2020 på www.udir.no

UNESCO, UNICEF, WHO. Considerations for school-related public health measures in the context of COVID-19**.** Annex to Considerations in adjusting public health and social measures in the context of COVID-19. September 2020. https://www.who.int/publications/i/item/considerations-for-school-related-public-health-measures-in-the-context-of-covid-19

Norwegian Institute of Public Health, 4 September 2020: Risk of COVID-19 infection in schools and in kindergartens: https://www.fhi.no/publ/2020/risiko-for-smitte-av-covid-19-pa-skoler-og-i-barnehager/
